# Association between metabolic syndrome and endometrial cancer risk: a systematic review and meta-analysis of observational studies

**DOI:** 10.18632/aging.103247

**Published:** 2020-05-22

**Authors:** Lan Wang, Zhen-Hua Du, Jia-Ming Qiao, Song Gao

**Affiliations:** 1 Department of Obstetrics and Gynecology, Shengjing Hospital of China Medical University, Shenyang, China

**Keywords:** endometrial cancer, metabolic syndrome, meta-analysis, risk

## Abstract

Existing evidence has revealed inconsistent results on the association between metabolic syndrome (MetS) and endometrial cancer (EC) risk. Herein, we aim to better understand this association. Systematic searches of PubMed, EMBASE, and Web of Science through 12 December 2019 were conducted. Observational studies that provided risk estimates of MetS and EC risk were eligible. The quality of the included studies was judged based on the Newcastle–Ottawa scale. Summary odds ratios (ORs) and 95% confidence intervals (CIs) were calculated using a random-effects model. Six studies, comprising 17,772 EC cases and 150,371 participants were included. MetS, diagnosed according to the criteria of the National Cholesterol Education Program—Third Adult Treatment Panel, was associated with an increased risk of EC (OR: 1.62; 95% CI = 1.26–2.07) with substantial heterogeneity (I^2^ = 78.3%). Furthermore, we found that women with MetS, diagnosed according to the criteria of the International Diabetes Federation, had a significantly higher risk of EC compared to healthy controls (OR: 1.45; 95% CI = 1.16–1.81; I^2^ = 64.6%). Our findings were generally consistent with the main results in the majority of prespecified subgroups, as well as in sensitivity analyses. In conclusion, MetS is associated with EC risk.

## INTRODUCTION

Endometrial cancer (EC) is the most common gynecologic cancer in developed countries, and its incidence and associated mortality are on the rise [1]. In 2018, more than 380,000 new cases and approximately 90,000 deaths worldwide were from EC, making it responsible for 4.4% of cancer cases and 2.1% of deaths due to cancer in women [2]. The prevalence of EC varies in different regions [3], which may be attributed to disparities in the incidence of obesity, as well as other important risk factors, such as aging, early age at menarche, late-onset menopause, nulliparity, and hormone replacement therapy use [4].

As the worldwide burden of EC continues to increase, interest is growing in the development of early preventive strategies for women at increased risk [5]. Metabolic syndrome (MetS) is an aggregation of several metabolic abnormalities, which include obesity, insulin resistance, hypertension, and dyslipidemia [6]. Irrespective of the diagnostic criteria used, the world is currently facing a significant epidemic of MetS. More than 20% of adults in most Asia–Pacific countries are affected by MetS [7], and approximately one-third of the adult population in the United States has MetS [8]. Studies suggest that metabolic abnormalities may be important risk factors for the development of EC. According to the results of detailed epidemiological studies, obesity is one of the most important risk factors for EC [9], whereas other studies have reported that diabetes is also a risk factor for EC, independent of obesity [10–12]. In addition, other metabolic abnormalities, such as hypertension [13] and dyslipidemia [14], are also associated with increased EC risk.

Although there is a general understanding of the association between EC risk and metabolic abnormalities, the association with MetS has not been established. Two meta-analyses [15, 16] have linked MetS, diagnosed according to the criteria of the National Cholesterol Education Program—Third Adult Treatment Panel (NCEP-ATP III), to an increased risk of EC. However, these findings should be interpreted with caution due to several limitations. Firstly, the risk estimates provided by the studies included in the meta-analyses were not suitable in evaluating the association between MetS and EC risk. For example, a meta-analysis conducted by Esposito et al. included a study reporting the association between metabolic abnormalities (defined as at least one of the following: diabetes, hypertension, overweight/obesity, dyslipidemia), rather than MetS and EC risk. Secondly, moderate or substantial heterogeneity was observed in these two meta-analyses, but the potential sources of heterogeneity were not fully explored. Thirdly, there was no evaluation of the association between MetS according to its different definitions and EC risk. Fourthly, two studies with large sample sizes (n = 13061 [17]; n = 117074 [18]), examining the same research area, have been recently published after these two meta-analyses, suggesting that this association is worth investigating. Therefore, we performed a systematic review and meta-analysis of observational studies to identify associations between MetS, diagnosed according to different criteria, and EC risk.

## RESULTS

### Literature search

**Figure 1:**
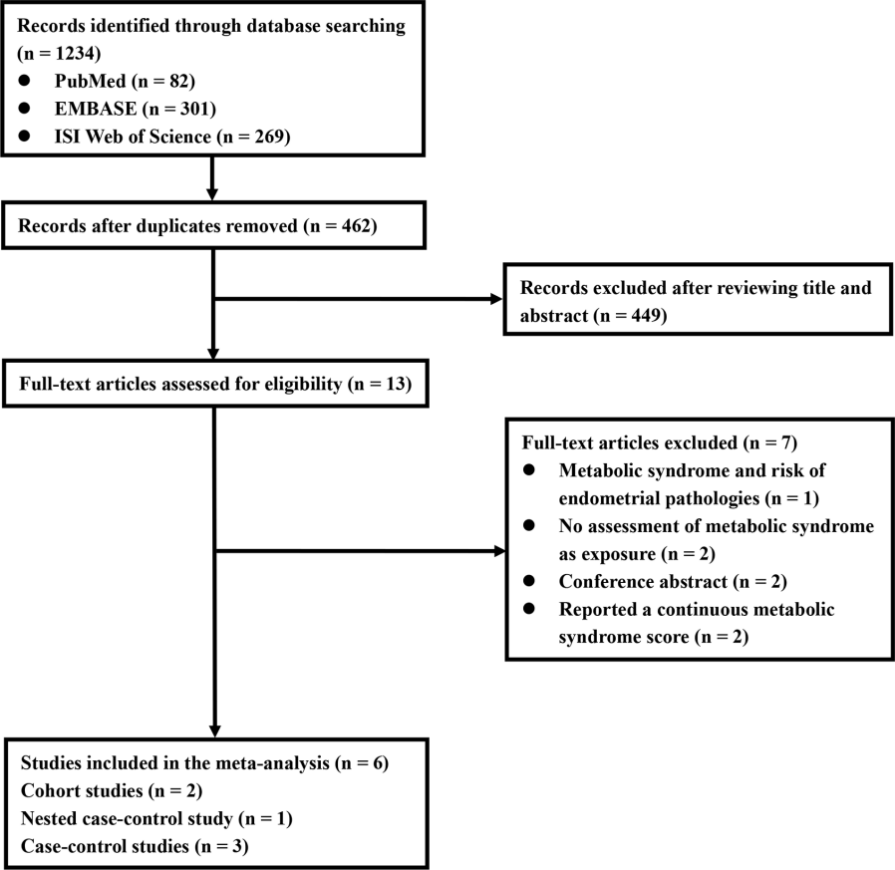
**Flowchart of included studies for the meta-analysis.**

As shown in Figure 1, a literature search identified 82, 269, and 301 potentially relevant records from PubMed, Web of Science, and EMBASE electronic bibliographic databases, respectively. A total of 462 records remained after the removal of duplicates. We excluded 449 records after screening titles and abstracts, and 13 articles were carefully scrutinized by reading the full text. Seven articles were excluded because of the following reasons: (i) the EC relevant outcome was not available (n = 1), (ii) MetS was not considered as the exposure (n = 2), (iii) the association between the per unit increase in the MetS score and the risk of EC was reported (n = 2), and (iv) the article was a conference abstract (n = 2). Finally, six studies were identified and included in our meta-analysis. Six studies [17–22] described the association between MetS, diagnosed according to NCEP-ATP III criteria, and EC risk. Five studies [17–20, 22] described the association between MetS, diagnosed according to the criteria of the International Diabetes Federation (IDF), and EC risk.

### Study characteristics and quality assessment

The characteristics of the included studies are summarized in Table 1. These studies were published between 2007 and 2019, and included two cohort studies [17, 21], one nested case-control study [19], and three case-control studies [18, 20, 22]. Three studies [17, 18, 22] were conducted in North America and three [19–21] in Europe. Five studies [17–19, 21, 22] recruited subjects from the general population, whereas the remaining study [20] recruited subjects from the hospital. The number of EC cases ranged from 20 to 16,323, with a total of 17,772 EC cases. The sample size varied from 830 to 117,074, with a total of 150,371 participants. According to the criteria of national or international scientific associations, federations, or organizations (including NCEP ATP III criteria, IDF criteria, the harmonized definition, and modifications based on these definitions), three studies [17, 19, 22] examined metabolic abnormalities through laboratory tests, physical examinations, and self-reported information. According to NCEP ATP III criteria, one study [21] defined MetS as the simultaneous use of three prescription drugs (antihypertensive, hypoglycemic, and hypolypemic drugs). According to NCEP ATP III and IDF criteria, one study [20] used self-reported information on the history of diabetes, drug-treated hypertension, drug-treated hyperlipidemia, and various measures of obesity to evaluate metabolic abnormalities. One study [18] used the criteria of the Clinical Modification of the International Classification of Diseases Revision 9 from inpatients/outpatients 1 to 3 years before EC diagnosis to identify metabolic abnormalities and to further define MetS according to NCEP ATP III and IDF criteria. The results and adjustment factors of the included studies are provided in Supplementary Table 1. Irrespective of the criteria used, five observational studies [17–20, 22] reported a positive association between MetS and EC risk. Four studies [17, 18, 20, 22] reported risk estimates after adjusting for confounders or stratifying by confounders. One study [19] controlled for the influence of the potential confounding factors on the results by matching.

**Table 1:** Characteristics of studies on the presence of metabolic syndrome and endometrial cancer risk.

**Study; location**	**Study design; study period**	**Study source**	**Mean age (or range) [year]**	**Cases/sample size**	**Diagnostic criteria for MetS**	**Determination of components of MetS**
Arthur et al. (2019); USA	Cohort; 1993-2017	Population-based	64.3	176/13061	NCEP ATP III, IDF, and modified NCEP ATP III (excluding WC)	Laboratory assays and anthropometric measurements
Trabert et al. (2015); USA	C/C; 1993-2007	Population-based	77	16323/117074	NCEP ATP III and IDF	ICD-9-CM codes from inpatient/outpatient diagnoses 1 to 3 years before case diagnosis
Friedenreich et al. (2011); Canada	C/C; 2002-2006	Population-based	58	515/1477	Harmonized definition, NCEP ATP III, IDF, and modified IDF (WC ≥88cm)	Laboratory assays and anthropometric measurements
Rosato et al. (2011); Italy	C/C; 1992-2006	Hospital-based	19-79	454/1252	NCEP ATP III and IDF	Self-reported history of diabetes, drug-treated hypertension, drug-treated hyperlipidemia and various measures of central obesity
Russo et al. (2008); Italy	Cohort; 1999-2005	Population-based	40+	20/16677	NCEP ATP III	Simultaneously prescribed with antihypertensive, hypolypemic and hypoglycemic drugs
Cust et al. (2007); Europe	N-C/C; 1992-2004	Population-based	56.9	284/830	NCEP ATP III and IDF	A combination of measured and self-reported data

C/C, case-control; ICD-9-CM, Clinical Modification of the International Classification of Diseases revision 9; IDF, International Diabetes Federation; MetS, metabolic syndrome; N-C/C, nested case-control; NCEP ATP-III, Adult Treatment Panel III of the National Cholesterol Education Program; WC, waist circumference.

Summaries of the assessments of the methodological quality of the included studies are shown in Supplementary Tables 2 and 3. In general, two perspective studies [17, 19] and two case-control studies [18, 22] were of high quality. The mean quality assessment score was 7, with a range from 5 to 8. In addition, three studies [17, 19, 22] were considered to have a low risk of bias.

### MetS and EC risk

**Figure 2:**
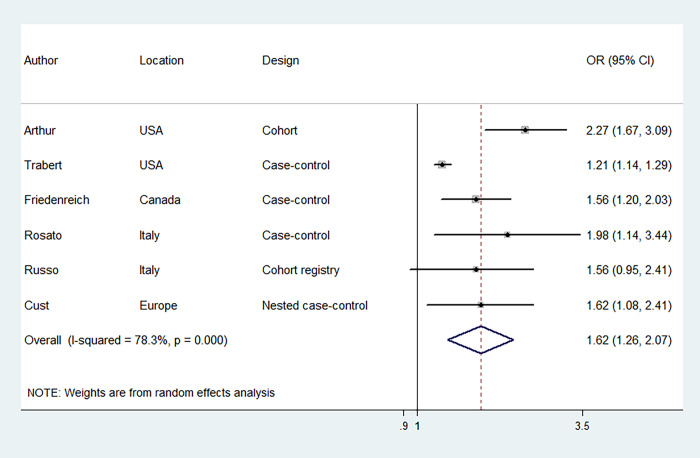
**Forest plots (random effect model) of meta-analysis on the association between the presence of metabolic syndrome based on the National Cholesterol Education Program—Third Adult Treatment Panel criteria and endometrial cancer risk.** Squares indicate study-specific ORs (size of the square reflects the study-specific statistical weight); horizontal lines indicate 95% CIs; diamond indicates the summary OR with its 95% CI. OR: odds ratio; CI: confidence interval.

**Figure 3:**
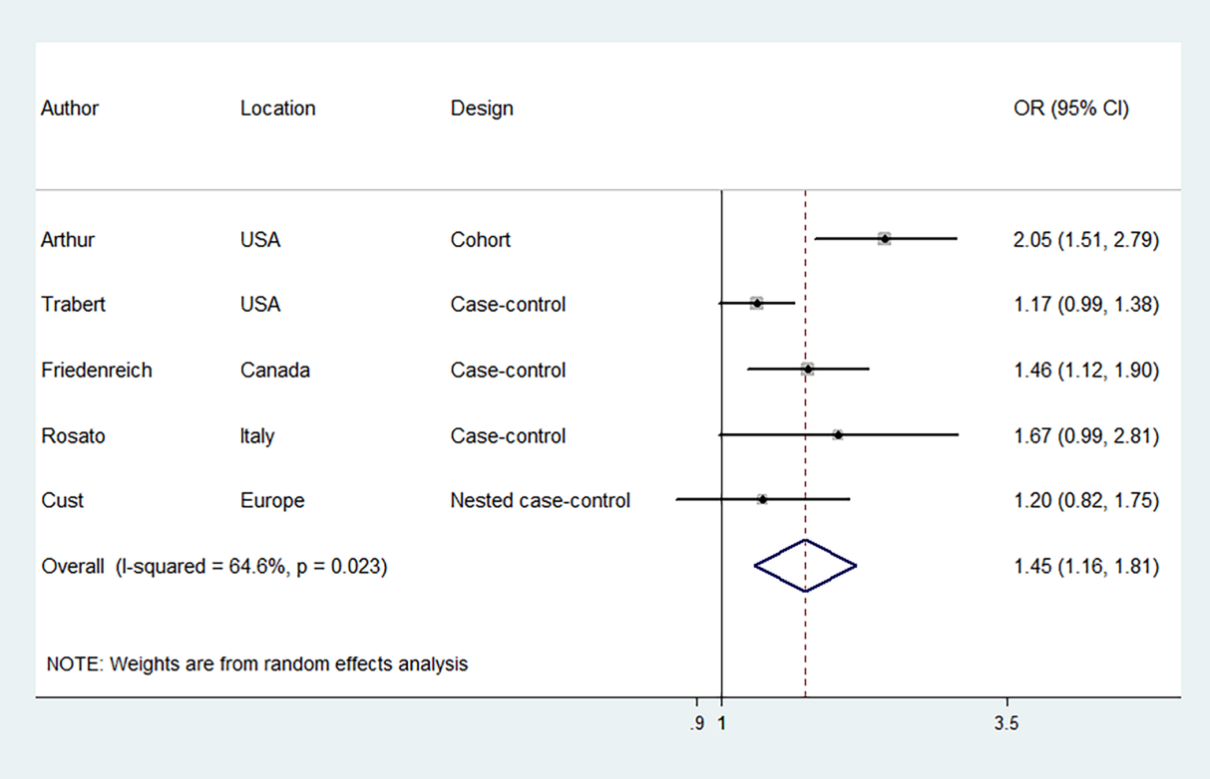
**Forest plots (random effect model) of meta-analysis on the association between the presence of metabolic syndrome based on the International Diabetes Federation criteria and endometrial cancer risk.** Squares indicate study-specific ORs (size of the square reflects the study-specific statistical weight); horizontal lines indicate 95% CIs; diamond indicates the summary OR with its 95% CI. OR: odds ratio; CI: confidence interval

Six studies were included to evaluate the association between MetS, diagnosed according to NCEP-ATP III criteria, and EC risk, involving 17,772 EC cases and 150,371 participants. Figure 2 illustrates that women with MetS had a higher risk of EC compared to those without MetS [odds ratio (OR) = 1.62, 95% confidence interval (CI): 1.26–2.07] with significant statistical heterogeneity between the included studies (I^2^ = 78.3%). The risk estimates were not significantly altered when individual studies were removed one at a time (Supplementary Figure 1). We further evaluated the association between MetS, diagnosed according to IDF criteria, and EC risk. Five studies were included, involving 17,752 EC cases and 133,694 participants. Likewise, the results revealed that MetS, diagnosed according to IDF criteria, was associated with an increased risk of EC (OR = 1.45, 95% CI: 1.16–1.81) (Figure 3). There was moderate statistical heterogeneity between the included studies (I^2^ = 64.6%) in the summary analysis. The risk estimates were not significantly altered when individual studies were removed one at a time (Supplementary Figure 2).

### Subgroup analyses and meta-regression

In subgroup analyses conducted according to geographical location, type of design, determination of the individual components of MetS, and risk of bias, the results were consistent in showing a positive association between MetS, diagnosed according to NCEP-ATP III criteria, and EC risk. We were unable to observe significant associations between MetS, diagnosed according to IDF criteria, and EC risk in some stratifications, probably because of the limited number of studies. Meta-regression analyses indicated that all selected study characteristics were not significantly associated with heterogeneity. The relevant results are presented in Table 2.

**Table 2:** Subgroup analyses and meta-regression for the association between the presence of metabolic syndrome and endometrial cancer risk.

	**NCEP ATP III**				**IDF**			
	**No. of studies**	**SOR (95% CI)**	***I^2^* (%)**	P^m^^*^	**No. of studies**	**SOR (95% CI)**	***I^2^* (%)**	P^m^^*^
Overall	6	1.62 (1.26-2.07)	78.3		5	1.45 (1.16-1.81)	64.6	
Subgroup								
Geographical location				0.76				0.81
North America	3	1.59 (1.10-2.28)	89		3	1.49 (1.08-2.04)	80.6	
Europe	3	1.68 (1.28-2.19)	0		2	1.35 (0.99-1.83)	0.9	
Type of design				0.22				0.52
perspective studies	3	1.87 (1.46-2.40)	22.5		2	1.59 (0.94-2.68)	78.4	
Retrospective studies	3	1.42 (1.10-1.84)	68		3	1.32 (1.08-1.60)	34.7	
Determination of components of MetS				0.25				0.50
LA and AM	3	1.79 (1.40-2.29)	44.1		3	1.55 (1.16-2.07)	61	
Proxy indicators	3	1.41 (1.06-1.87)	51.1		2	1.28 (0.95-1.73)	38.4	
Risk of bias				0.25				0.50
Low	3	1.79 (1.40-2.29)	44.1		3	1.55 (1.16-2.07)	61	
High	3	1.41 (1.06-2.07)	51.1		2	1.28 (0.95-1.73)	38.4	
Adjust age				0.92				NA
Yes	5	1.63 (1.24-2.16)			5	1.45 (1.16-1.81)	64.6	
No	1	1.56 (0.98-2.48)	NA		0			
Adjust race/ethnicity				0.87				0.84
Yes	2	1.63 (0.88-3.01)	93.5		2	1.52 (0.88-2.64)	89.9	
No	4	1.62 (1.34-1.95)	0		3	1.41 (1.15-1.72)	0	
Adjust education				0.07				0.07
Yes	2	2.20 (1.68-2.88)	0		2	1.94 (1.49-2.53)	0	
No	4	1.39 (1.15-1.67)	51.4		3	1.24 (1.09-1.41)	0	
Adjust smoking status				0.86				0.84
Yes	2	1.63 (0.88-3.01)	93.5		2	1.52 (0.88-2.64)	89.9	
No	4	1.62 (1.34-1.95)	0		3	1.41 (1.15-1.72)	0	
Adjust physical activity				0.13				0.09
Yes	1	2.27 (1.67-3.09)	NA		1	2.05 (1.51-2.79)	NA	
No	5	1.45 (1.19-1.76)	55.1		4	1.27 (1.11-1.45)	4.5	
Adjust HRT use				0.13				0.08
Yes	3	1.88 (1.45-2.43)	40.7		3	1.70 (1.35-2.13)	25.9	
No	3	1.32 (1.09-1.60)	34.4		2	1.17 (1.01-1.37)	0	
Adjust OC use				0.07				0.07
Yes	2	2.20 (1.68-2.88)	0		2	1.94 (1.49-2.53)	0	
No	4	1.39 (1.15-1.67)	51.4		3	1.24 (1.09-1.41)	0	
Adjust menopausal status				0.08				0.35
Yes	3	2.00 (1.60-2.50)	0		3	1.62 (1.15-2.29)	56.8	
No	3	1.35 (1.11-1.65)	54.7		2	1.27 (1.03-1.58)	48.3	
Adjust age at menarche				0.79				0.77
Yes	2	1.63 (1.29-2.07)	0		2	1.50 (1.19-1.90)	80.3	
No	4	1.60 (1.13-2.25)	83.3		3	1.41 (0.98-2.03)	0	
Adjust gravidity				0.90				0.99
Yes	1	1.56 (1.20-2.03)	NA		1	1.46 (1.12-1.90)	NA	
No	5	1.65 (1.20-2.25)	80.6		4	1.45 (1.07-1.97)	72.6	
Adjust parity				0.57				0.69
Yes	1	1.98 (1.14-3.44)	NA		1	1.67 (0.99-2.81)	NA	
No	5	1.58 (1.22-2.05)	80.5		4	1.42 (1.10-1.83)	71.9	
Adjust overweight/obesity				0.11				0.08
Yes	2	1.31 (1.02-1.68)	49.6		2	1.17 (1.01-1.37)	0	
No	4	1.81 (1.48-2.22)	21.1		3	1.70 (1.35-2.13)	25.9	

AM, anthropometric measurements; HRT, hormone replacement therapy; IDF, International Diabetes Federation; LA, laboratory assays; MetS, metabolic syndrome; NA not applicable; NCEP ATP-III, Adult Treatment Panel III of the National Cholesterol Education Program; OC, oral contraceptive; SOR, summary odds ratio.

## DISCUSSION

Irrespective of the diagnosis of MetS (NCEP-ATP III or IDF criteria), we found that MetS was positively associated with the risk of EC in our meta-analysis of observational studies. These findings were largely consistent across subgroups defined by various study characteristics and stable in sensitivity analyses. To our best knowledge, no meta-analysis has been performed to evaluate the association between MetS, diagnosed according to IDF criteria, and EC risk.

The mechanistic understanding by which MetS promotes EC is unclear, although it may be attributed to abnormal fat metabolism, chronic inflammation, hyperglycemia, and hyperinsulinemia [23]. A previous study revealed that adiponectin, which is secreted mainly by white adipose tissue, can activate the liver kinase B1–AMP–activated protein kinase (LKB1–AMPK) signaling pathway to inhibit the proliferation and invasion of EC cells by binding to adiponectin receptors [24]. Elevated leptin levels, which are encoded by the obesity gene, have been shown to promote the proliferation and invasion of EC cells by activating a variety of signaling pathways [23]. In addition, studies have demonstrated that adipose-derived inflammatory cytokines, such as IL-6 [25] and TNF-α [23], are involved in the development and progression of EC. Elevated serum glucose levels can enhance glycolysis through the AMPK signaling pathway, thereby resulting increased invasiveness of EC cells [23]. Glycolysis cannot only generate much-needed energy for tumor cells, but also produce numerous metabolic intermediates, which can be used by tumor cells in the synthesis of biological macromolecules [23]. In a previous study [26], metformin was found to suppress EC cell proliferation, which may be attributed to the activation of the AMPK signaling pathway. However, the use of metformin in the treatment of EC is still controversial, and further research is warranted to identify critical molecules and new drugs. Insulin can activate the phosphoinositide 3–kinase/protein kinase B or mitogen–activated protein kinase/extracellular signal–regulated kinase signaling pathway by binding to insulin receptor/insulin-like growth factor-1 receptor, thereby promoting epithelial-mesenchymal transition and increasing the proliferation and invasion of EC cells [27]. Furthermore, a recent study has reported that cholesterol can activate the transcriptional activity of EC cells and promote the proliferation of EC cells [28].

In addition to the elevated burden of MetS, metabolic abnormalities, such as obesity and diabetes, also contribute to an increased risk of developing EC. Improvements in the overall metabolic state, especially weight maintenance and blood glucose control, have enormous clinical implications for both MetS and EC. Previous studies have linked EC risk to metabolic abnormalities such as obesity, diabetes, and hypertension. These disorders are collectively known as the metabolic triad of EC [23]. There is compelling evidence indicating that being overweight or obese increases the risk of EC. According to a large number of epidemiological studies performed worldwide, obesity has been identified as the convincing cause of EC by the World Cancer Research Fund International Continuous Update Project [9], and it is responsible for nearly 40% of all EC cases in the Western world [29]. Three dose-response meta-analyses [30–32] showed that an increase in the body mass index (BMI) by 5 kg/m^2^ can increase the risk of a women developing EC by 54%, 59%, and 60%, respectively. The evidence for abdominal fatness and weight gain was weaker than that using the BMI as the measure of body fatness, although it still supported a positive association between overall body fatness and EC risk [33]. Another metabolic abnormality, diabetes, has also been found to be associated with EC risk, independent of obesity [11, 12]. Two meta-analyses [34, 35] quantitatively summarized the results of published observational studies reporting a greater risk of EC in subjects with diabetes compared with those without diabetes. In addition, the results of subgroup analysis according to the type of diabetes supported a positive association between type 1 and type 2 diabetes and EC [34]. The contribution of hypertension to EC has also been established. A previous meta-analysis [36] involving 300,598 participants and 28,385 EC cases has reported an increased risk of EC among patients with hypertension. There was no significant change in the magnitude and direction of the association between hypertension and EC risk among studies after adjusting for the BMI. However, few studies have examined the association between serum lipid profiles and EC risk. Two prospective studies [14, 19] reported a positive association between low serum high-density lipoprotein-cholesterol (HDL-C) levels and high serum triglyceride levels and EC risk, although the association between serum triglyceride levels and EC risk was not statistically significant when the BMI was included in the model [14].

Various heterogeneous factors can contribute to the risk of EC. For example, different regions of the world have distinct differences in culture, economy, and education that may affect the occurrence of EC. Although subgroup analyses by region indicated that MetS significantly increased the risk of EC in women in North America and Europe, it was difficult to determine which factors contributed to the observed positive association due to the unavailability of information. Therefore, several area-related factors, namely the economy and education, need to be considered in further research. Consistent with two previous meta-analyses [15, 16], there was considerable heterogeneity in our summary analyses. Differences in various characteristics among the included studies may have influenced the heterogeneity, including the type of design, determination of metabolic abnormalities, risk of bias, and adjustment factors. As shown in subgroup analyses, irrespective of the diagnosis of MetS (NCEP-ATP III or IDF criteria), we found that the heterogeneity was reduced among studies using the results of laboratory assays and anthropometric measurements to determine the metabolic abnormalities or proxy indicators, suggesting that the methods used to identify the metabolic abnormalities may be responsible for the significant heterogeneity in our analysis. The metabolic abnormalities were not directly identified by the results of laboratory assays and anthropometric measurements in the three included studies [18, 20, 21], but replaced by the proxy indicators. In a study [21] using linked pharmaceutical and cancer registry data, Russo et al. reported no association between MetS defined according to combined prescription patterns and EC risk. Trabert et al. identified the metabolic abnormalities based on outpatient/inpatient data rather than direct measurements of blood pressure; the levels of fasting serum triglycerides, HDL-C, fasting plasma glucose, and waist circumference, and found a positive association between MetS and EC risk [18]. We found that the heterogeneity was significantly reduced after excluding the study by Trabert et al., irrespective of whether it was based on NCEP ATP III (I^2^ = 0.7%) or IDF (I^2^ = 42%) criteria. Another study [20] showed that the EC risk was significantly increased for subjects with MetS diagnosed according to self-reported histories of diabetes, drug-treated hypertension, drug-treated hyperlipidemia, and obesity. In addition, according to NCEP ATP III criteria, the results of the present meta-analysis indicate that a proportion of the observed heterogeneity may be explained by differences in study design.

The present meta-analysis has several strengths. Firstly, our findings are in agreement with two previous meta-analyses that reported a positive association between MetS and EC risk. However, with a careful screening process and a large sample size, we included six observational studies investigating the association between MetS and EC risk. Therefore, our strong statistical power could detect positive associations. In addition, our results were consolidated by further subgroup and sensitivity analyses. Secondly, the positive association of MetS with EC risk was observed not only in studies using NCEP-ATP III criteria to diagnose MetS but also in those using IDF criteria. To our best knowledge, this is the first meta-analysis to quantitatively evaluate the association between MetS, diagnosed according to different criteria, and EC risk. Thirdly, there was significant heterogeneity in our study. Thus, detailed subgroup and univariate meta-regression analyses were used to assess whether results varied according to key study characteristics.

Our findings should be interpreted with caution, as there are several limitations. Firstly, clinical and methodological heterogeneity is always a concern for all meta-analyses, particularly for meta-analyses of observational studies [37]. Irrespective of the diagnostic criteria used for MetS, there was considerable heterogeneity in our meta-analysis. Although meta-regression analyses were used, the limited number of included studies may have restricted the power of meta-regression in exploring the sources of heterogeneity. Secondly, cohort, case-control, and nested case-control studies were all represented in our meta-analysis, and the methodological differences in study designs may have biased the results because of significant variations in analyses. Considering that half of the included studies were case-control studies, our results may have been subjected to recall bias. Thirdly, although the included studies attempted to control for known risk factors and we also extracted maximally adjusted risk estimates, the possibility of residual confounding could not be excluded, given that our findings originate from observational studies where residual confounding always exists [37]. In addition, single-point measurements increase the chance of random measurement errors, which may lead to attenuation of the reported associations [16]. Finally, we did not assess publication bias using the funnel plot or Egger regression test, as they have insufficient power in cases of limited studies (n < 10). Thus, we cannot eliminate the possibility that our summary results are driven by publication bias. Nevertheless, all meta-analyses are subject to publication bias due to the possibility of under-reporting negative results or failing to identify the ‘grey literature’ (i.e., the articles that are not published formally by publishers, including conference proceedings, magazine articles, and government papers) [38].

In conclusion, the results of this meta-analysis suggest MetS associates with an increased risk of EC. Considering the limitations of this meta-analysis, further studies with perspective designs are warranted to confirm our findings, with an emphasis on determining the metabolic abnormalities and adjusting for several key confounding factors (e.g., overweight/obesity or BMI/WC).

## MATERIALS AND METHODS

### Literature search

The review protocol and reporting of the data were conducted according to the Meta-analysis of Observational Studies in Epidemiology [39] and Preferred Reporting Items for Systematic Reviews and Meta-analyses guidelines [40]. We conducted comprehensive searches in PubMed, EMBASE, and Web of Science to identify all potentially relevant articles up to 12 December 2019. The keywords used in the literature searches were as follows: (“metabolic syndrome” OR “insulin resistance syndrome” OR “syndrome X” OR “dysmetabolic syndrome” OR “plurimetabolic syndrome” OR “cardiometabolic syndrome”) AND (“endometrial cancer” OR “endometrium cancer” OR “endometrial carcinoma” OR “endometrium carcinoma” OR “endometrial neoplasm”). In addition, the reference lists of all included articles, as well as related reviews and meta-analyses, were further examined for additional eligible articles.

### Selection criteria

The articles satisfying all of the following criteria were included in our meta-analysis: (1) used a cohort, case-control, nested case-control, or cross-sectional study design; (2) investigated the association between MetS and EC risk; and (3) reported risk estimates (relative risks, ORs, hazard ratios, and standardized incidence ratios) and corresponding 95% CIs. The articles satisfying any of the following criteria were excluded: (1) investigated the association between metabolic abnormalities and EC risk; (2) provided risk estimates of EC per unit increase in the MetS score (generated by adding individual z-scores computed for body mass index; blood pressure; and the levels of glucose, cholesterol, and triglycerides), because the estimates did not represent the risk of EC in subjects with MetS compared to those without MetS; and (3) presented results as conference abstracts, considering that the results may vary between meeting presentation and peer-reviewed publication [37]. Two researchers examined the titles and abstracts in accordance with the established inclusion criteria to exclude ineligible studies, and then read the full texts to further exclude ineligible studies. Any discrepancies were resolved by discussion.

### Data extraction and quality assessment

Two researchers independently extracted the relevant information from the studies that satisfied all of the eligibility criteria. The extracted information was as follows: first author’s family name, publication year, study location, study design, study period, study source, mean age (or range), number of participants, number of EC cases, diagnostic criteria for MetS, determination of metabolic abnormalities, maximally adjusted risk estimates and 95% CIs (presence versus absence of MetS), and covariates matched in the study design or adjusted in the statistical analysis. Discrepancies in extracted data between researchers were resolved by consensus or discussion with a third author.

Two researchers independently performed quality assessment of the included observational studies using the Newcastle–Ottawa scale. This scale is comprised of eight items, which fall into three domains: selection of the population (ranging from 0 to 4 points), comparability of the groups (ranging from 0 to 2 points), and assessment of the outcome (ranging from 0 to 3 points). We considered a study to be of high quality when the total score was ≥7 points. In addition, studies that scored full marks in at least two domains were considered to have a low risk of bias. Quality assessment measured the strength of the scientific evidence; however, it was not used to determine which studies should be included [41].

### Statistical analysis

We summarized the risk estimate from each study using a random-effects model. The OR was used to assess the association between MetS and EC risk. The average of the natural logarithm of the ORs was estimated, and the OR from each study was weighted by the inverse of its variance. The I^2^ statistic (I^2^ >75.0%, 50.0–75.0% and <50% indicating substantial, moderate, and low heterogeneity, respectively) was used to quantitatively evaluate statistical heterogeneity [42]. Subgroup and meta-regression analyses were performed to explore sources of study heterogeneity and the significance of the differences in the ORs by different subgroups, including geographical location (North America versus Europe), type of design (perspective studies versus retrospective studies), determination of metabolic abnormalities (laboratory assays and anthropometric measurements versus proxy indicators), risk of bias (low versus high), and adjustment of covariates (including age, race/ethnicity, education level, smoking status, physical activity, hormone replacement therapy use, oral contraceptive use, menopausal status, age at menarche, gravidity, parity, and overweight/obesity). Sensitivity analyses assessed whether the overall OR could be significantly affected by omitting a single study. We did not test publication bias using formal statistical tests, because they have limited power in cases of <10 studies [37]. STATA software (version 11.2, StataCorp LP, College Station, TX, USA) was used for all data analyses. A two-tailed *P* <0.05 was considered statistically significant.

## Supplementary Material

Supplementary Figures

Supplementary Tables
