# Supplementary material for: Association between metabolic syndrome and endometrial cancer risk: a systematic review and meta-analysis of observational studies
Source: Aging (Albany NY). 2020 May 22;12(10):9825–39. doi: 10.18632/aging.103247 (PMC7288955; doi:10.18632/aging.103247)
Supplement: Supplementary Figures [file aging-12-103247-s001..pdf]

SUPPLEMENTARY FIGURES

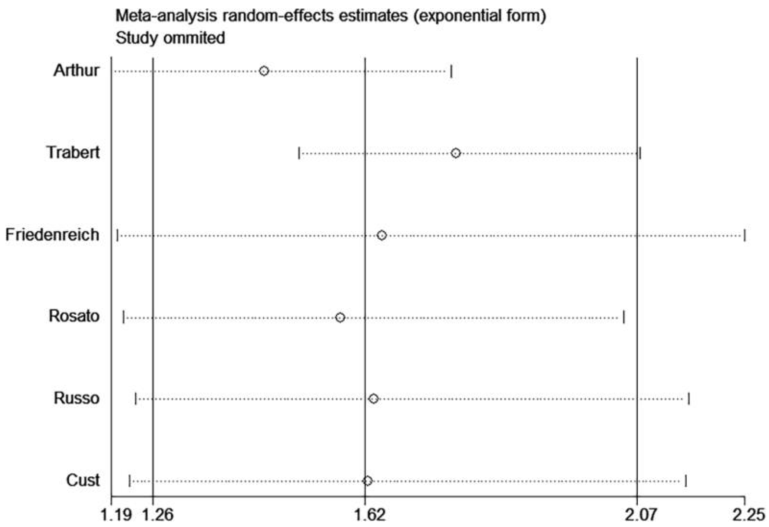

Supplementary Figure 1. Sensitivity analysis of studies investigating the association between the presence of metabolic syndrome diagnosed based on the National Cholesterol Education Program—Third Adult Treatment Panel criteria and endometrial cancer risk.

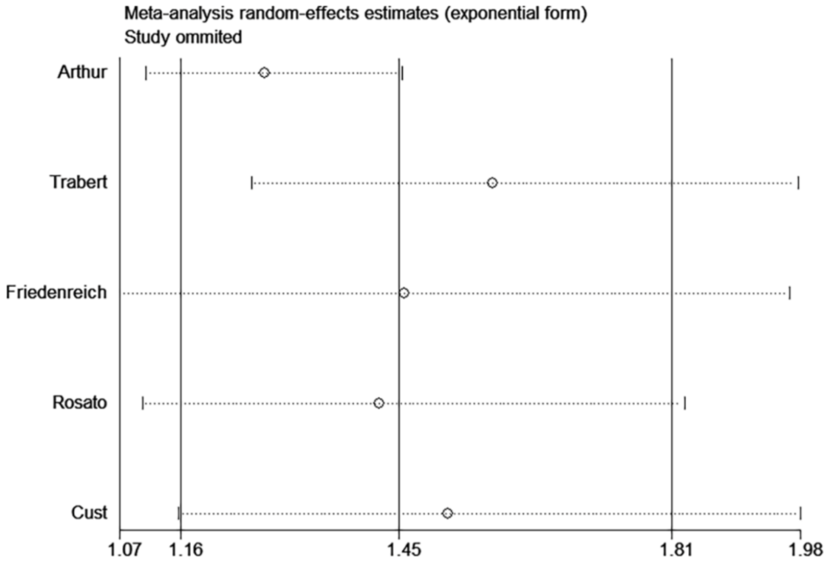

Supplementary Figure 2. Sensitivity analysis of studies investigating the association between the presence of metabolic syndrome diagnosed based on the International Diabetes Federation criteria and endometrial cancer risk.
