# Supplementary material for: Association between metabolic syndrome and endometrial cancer risk: a systematic review and meta-analysis of observational studies
Source: Aging (Albany NY). 2020 May 22;12(10):9825–39. doi: 10.18632/aging.103247 (PMC7288955; doi:10.18632/aging.103247)
Supplement: Supplementary Tables [file aging-12-103247-s002..pdf]

SUPPLEMENTARY TABLES

Supplementary Table 1. Outcomes and covariates of the studies included in the meta-analysis.

Study	Risk estimates (95% CI)			Adjustment factors
Arthur et al. (2019)	NCEP ATP III	Age	< 65 years: 2.55 (1.66-3.92)<br>≥ 65 years: 1.92 (1.24-2.98)	With the exception of the stratifying variable, models were adjusted for age, pack-years of smoking, alcohol intake, physical activity, hormone therapy use, oral contraceptive use, menopausal status, education, ethnicity, allocation to the observational study or intervention/placebo/ control arm of each clinical trial
		Smoking	never: 1.73 (1.16-2.59)<br>ever: 3.45 (1.10-5.69)	
		Hormone therapy use	never: 2.38 (1.62-3.49)<br>ever: 1.83 (1.03-3.25)	
		Hormone therapy use (excluding women from the hormone therapy intervention)	never: 2.61 (1.71-3.99)<br>ever: 1.72 (0.88-3.35)	
		BMI	< 25: 1.83 (0.61-5.46)<br>25-29.9: 1.31 (0.62-2.76)<br>≥ 30: 1.57 (1.04-2.36)	
		Type I: 2.49 (1.77-3.50)<br>Total: 2.27 (1.67-3.09)		Age, pack-years of smoking, alcohol intake, physical activity, hormone therapy ever, oral contraceptive ever, menopausal status, education, ethnicity, family history of endometrial cancer and allocation to the observational study or intervention/placebo/control arm of each clinical trial
	IDF	Type I: 2.22 (1.55-3.12)<br>Total: 2.05 (1.51-2.79)		
	Modified NCEP ATP III (excluding WC): 1.34 (0.97-1.84)			
Trabert et al. (2015)	NCEP ATP III	Endometrioid grade 1-2: 1.42 (1.31-1.53)<br>Endometrioid grade 3: 1.40 (1.21-1.61)<br>Adenocarcinoma: 1.41 (1.27-1.56)<br>Serous: 1.41 (1.19-1.67)<br>Clear cell: 1.46 (1.06-2.02)<br>Mucinous: 1.69 (1.14-2.50)<br>Carcinosarcoma: 1.14 (0.94-1.38)<br>Sarcoma: 1.34 (0.94-1.92)<br>Other: 1.31 (1.08-1.58)<br>Type I: 1.41 (1.32-1.50)<br>Type II: 1.42 (1.22-1.65)<br>Total: 1.21 (1.14-1.29)		Diagnosis date, age, race/ethnicity, registry area, tobacco use, and additionally adjusted for overweight/obesity for all cases
	IDF	Endometrioid grade 1-2: 2.09 (1.83-2.40)<br>Endometrioid grade 3: 2.02 (1.57-2.59)<br>Adenocarcinoma: 1.99 (1.67-2.38)<br>Serous: 2.29 (1.74-3.01)<br>Clear cell: 2.03 (1.15-3.60)<br>Mucinous: 4.06 (2.36-6.99)<br>Carcinosarcoma: 1.58 (1.12-2.24)<br>Sarcoma: 2.17 (1.22-3.85)<br>Other: 2.34 (1.72-3.17)<br>Type I: 2.03 (1.83-2.26)<br>Type II: 2.24 (1.74-2.88)<br>Total: 1.17 (0.99-1.38)		
Friedenreich et al. (2011)	Harmonized definition	Pre/perimenopausal: 1.96 (1.09-3.50)		Age, age at menarche, number of pregnancies ≥ 20 weeks gestation
		Postmenopausal: 1.39 (1.03-1.89)		Age, age at menarche, number of pregnancies ≥ 20 weeks gestation, hormone therapy use type
		BMI < 25: 1.84 (0.92-3.67)<br>BMI 25-29.9: 2.04 (1.33-3.11)<br>BMI > 30: 1.71 (1.13-2.58)		Age, age^2, age at menarche, number of pregnancies ≥ 20 weeks gestation, hormone therapy use type
		Total: 1.53 (1.17-2.00)		Age, age at menarche, number of pregnancies ≥ 20 weeks gestation, hormone therapy use type, adiponectin, insulin, and leptin
	NCEP ATP III: 1.56 (1.20-2.03)<br>IDF: 1.46 (1.12-1.90)<br>modified IDF (WC ≥88cm): 1.66 (1.26-2.19)			

Rosato et al. (2011)	NCEP ATP III: 1.98 (1.14-3.44)	Age, study center, year of interview, education, age at menarche, parity, menopausal status, oral contraceptive use, and hormone replacement therapy use
	IDF: 1.67 (0.99-2.81)	
Russo et al. (2008)	NCEP ATP III: 1.56 (0.95-2.41)	No adjustment
Cust et al. (2007)	NCEP ATP III: 1.62 (1.08-2.41)	The analysis was matched on study center, baseline menopausal status, age at blood collection, time of day of blood collection, fasting status, and in premenopausal women, phase of menstrual cycle; and the model was additionally adjusted for waist circumference
	IDF: 1.20 (0.82-1.75)	

BMI, body mass index; CI, confidence interval; IDF, International Diabetes Federation; MetS, metabolic syndrome; NCEP ATP-III, Adult Treatment Panel III of the National Cholesterol Education Program.

Supplementary Table 2. Results of the critical evaluation of included studies using the Newcastle–Ottawa Quality Assessment Scale for cohort studies

	Selection				Comparability	Outcome			
**Study**	**Representativeness of exposed cohort ☆**	**Selection of non-exposed cohort ☆**	**Exposure ascertainment ☆**	**No history of disease ☆**	**Comparable on confounders ☆☆**	**Outcome Assessment ☆**	**Adequate follow-up (≥10y) ☆**	**Loss to follow-up rate (≤20%) ☆**	**Total Score**
Arthur et al. (2019)	☆	☆	☆	☆	☆☆	☆	☆		8
Russo et al. (2008)	☆	☆	☆	☆		☆			5

Supplementary Table 3. Results of the critical evaluation of included studies using the Newcastle–Ottawa Quality Assessment Scale for case-control studies.

	Selection				Comparability	Outcome			
**Study**	**Adequate case definition ☆**	**Representativeness of cases ☆**	**Selection of controls ☆**	**Definition of controls ☆**	**Comparable on confounders ☆☆**	**Exposure ascertainment ☆**	**Ascertainment for cases and controls ☆**	**Non-response rate ☆**	**Total Score**
Trabert et al. (2015)	☆	☆	☆	☆	☆	☆	☆		7
Friedenreich et al. (2011)	☆		☆	☆	☆ ☆	☆	☆	☆	8
Rosato et al. (2011)	☆	☆		☆	☆	☆		☆	6
Cust et al. (2007)	☆	☆	☆	☆	☆ ☆	☆	☆		8
